# First detection of a reassortant G3P[8] rotavirus A strain in Italy: a case report in an 8-year-old child

**DOI:** 10.1186/s12985-019-1173-1

**Published:** 2019-05-15

**Authors:** Susanna Esposito, Barbara Camilloni, Sonia Bianchini, Giovanni Ianiro, Ilaria Polinori, Edoardo Farinelli, Marina Monini, Nicola Principi

**Affiliations:** 1 Paediatric Clinic, Department of Surgical and Biomedical Sciences, Università degli Studi di Perugia, Piazza Menghini 1, 06129 Perugia, Italy; 2 Microbiology Unit, Department of Medicine, Università degli Studi di Perugia, Perugia, Italy; 3 Department of Food Safety, Nutrition and Veterinary Public Health, Istituto Superiore di Sanità, Rome, Italy; 4 Università degli Studi di Milano, Milan, Italy

**Keywords:** Acute gastroenteritis, Diarrhoea, Gastrointestinal virus, G3P[8] rotavirus A, Rotavirus

## Abstract

**Background:**

Acute gastroenteritis (AGE) due to group A rotavirus (RVA) agent is one of the major causes of hospitalization in paediatric age. The G3P[8] RVA genotype has been usually considered as one of the major human genotypes, largely circulating in Asia, but showing low detection rates in the European countries. In recent years, the G3P[8] RVAs emerged also in Europe as a predominant genotype and the viral strains detected revealed high similarities with equine-like G3P[8] RVA strains, resulting in a new variant circulating in humans and able to cause AGE in the paediatric population.

**Case presentation:**

An 8-year-old boy was admitted to the Emergency Room because he had suffered from severe diarrhoea, vomiting, and high fever over the previous two days. Severe dehydration was evident based on low serum concentrations of potassium and sodium, low glycaemia, and pre-renal failure (creatinine 2.48 mg/dL, urea 133 mg/dL). Immunological tests were within normal range. Enzyme immunoassay for the detection of RV was positive, and a sample of faeces was collected in order to perform the molecular characterization of the viral strain. The phylogenetic trees revealed relatedness between the VP7 and VP4 genes of the G3P[8] RVA Italian strain (namely PG2) and those belonging to recent G3P[8] RVAs detected worldwide. The G3 VP7 belonged to the G3-I lineage and shared the highest nucleotide sequence identity (99.8%) with the equine-like G3 previously identified in other countries. The P [8] VP4 revealed a similar clustering pattern to that observed for the VP7. In addition, the molecular characterization of the 11 gene segments of strain PG2 revealed a G3-P[8]-I2-R2-C2-M2-A2-N2-T2-E2-H2 genomic constellation.

**Conclusions:**

This case shows the first detection in Italy of a reassortant G3P[8] RVA associated with a severe AGE, which is unusual in a school-age child without any known severe underlying problems. The findings reported in this paper highlight the importance of continuously monitoring the RVA strains circulating in paediatric age in order to detect novel viral variants able to spread in the general population.

## Background

Although available rotavirus vaccines have significantly reduced the total burden of rotavirus (RV) infection, acute gastroenteritis due to this infectious agent remains one of the most important causes of paediatric hospitalization in industrialized countries and death in the developing world [1]. The RV genome is composed of 11 segments of double-stranded RNA (dsRNA), encoding six structural (VP1–4, VP6, and VP7) and five or six non-structural proteins (NSP1-NSP5/6), as the segment 11 can encode two proteins (NSP5 and NSP6) [2]. Immunologic and genetic characteristics of VP6 protein are used to differentiate RV groups (or species). Among them, group A has the greatest importance as a cause of human infections. Group A rotavirus (RVA) has a binary classification system based on nucleotide sequence similarities of VP7 and VP4 genes, which determine the G- and P-genotype (i.e., glycoprotein, G protein and protease-cleaved spike protein, P protein, respectively). Since 2008, on the basis of whole genome sequencing, RVA can be divided in viruses with the Wa-like constellation (G1-P[8]-I1-R1-C1-M1-A1-N1-T1-E1-H1), DS-1-like constellation (G2-P[4]-I2-R2-C2-M2-A2-N2-T2-E2-H2), and AU-1-like constellation (G3-P[9]-I3-R3-C3-M3-A3-N3-T3-E3-H3) [2].

Currently 36 G-genotypes and 51 P-genotype have been detected, and at least 80 combinations have been identified in the RVA strains that infect humans and animals [2]. Although there are significant differences in geographical distribution, approximately 90% of the infections diagnosed in humans are due to six major human genotypes: G1P[8], G2P[4], G3P[8], G4P[8], G9P[8], and G12P[8] [3, 4]. However, the RVA genome can frequently evolve, and strains previously unknown or rarely detected can emerge. Point mutations, genetic reassortment, interspecies transmission, and genome rearrangement are the causes of new RVA emergence. Usually, human RVAs do not infect animals and vice versa. However, interspecies transmission and reassortment between animal and human strains can lead to RVAs that are able to spread successfully among humans [5]. Moreover, RVAs with significant genetic diversity may reduce the efficacy of presently available vaccines. Finally, the emergence of RVAs with particularly higher virulence is theoretically possible. All of these findings explain why continuous monitoring of genetic characteristics of RVA strains that are prevalent in a given geographic area is suggested [5].

In recent years, G3P[8] RVA has emerged as a predominant genotype in several countries, including Asia (Pakistan, Indonesia, Japan), South-America (Argentina), and Europe (Germany, Spain), and this high detection rates were, at least in part related to a new equine-like variant possessing a complete DS-1 like (genotype 2) genomic backbone (G3-P[8]-I2-R2-C2-M2-A2-N2-T2-E2-H2), and able to spread rapidly in the human population [6–9]. Since the establishment in January 2007 of the RV AGE surveillance in Italy, low rates of detection were observed for the G3P[8] genotype, and all the infections were linked to a typical human variant detected worldwide in association with the Wa-like (genotype 1) genomic constellation. In this paper, the first detection of an equine-like G3P[8] RVA in Italy is reported and discussed.

## Case presentation

### Clinical findings

An 8-year-old boy was admitted to the Emergency Room of Santa Maria della Misericordia hospital, Perugia, Italy, on February 10, 2018 because he had suffered from severe diarrhoea (> 6 stools per day), vomiting (3 episodes per day) and high fever (> 39.0 °C) over the previous two days. He did not have unusual travel, dietary, or animal contact and did not receive any doses of the RV vaccine. In the Emergency Room, the child was drowsy, body temperature was 38.7 °C, heart rate 170 beats/min, respiratory rate 50 breaths/min, and blood pressure 85/45 mmHg. Severe dehydration was evident. The weight was 2.5 kg lower than that evaluated several days before disease onset, revealing a 10% loss. The oral mucosa was very dry, eyes sunken and skin liftable in persistent folds. Moreover, no urine was found in the bladder after catheterization. Laboratory tests revealed low serum concentrations of both potassium (2.7 mEq/L) and sodium (128 mEq/L), confirming hypo electrolytic gastroenteritis. Glycaemia was lower than the normal (47 mg/dL). Kidney function tests showed a relevant pre-renal failure, with creatinine at 2.48 mg/dL and urea at 133 mg/dL. Severe acute gastroenteritis was diagnosed according to the Ruuska and Vesikari criteria for the definition of severity of RV diarrhoeal episodes [10], and the child was hospitalized.

After hospitalization, faecal samples for bacteria and virus identification were collected. Cultures for all the bacterial pathogens usually associated with acute severe gastroenteritis were negative, while an enzyme immunoassay for the detection of RVA (Ridascreen® Rotavirus, R-Biopharm AG, Germany) was positive. A sample of faeces was used for reverse transcription-polymerase chain reaction and nucleotide sequencing of the infecting RV. Moreover, tests were performed to evaluate host immune system function, including total immunoglobulin concentration, neutrophil function and lymphocyte immunophenotyping; however, no abnormalities were revealed.

Intravenous fluids were immediately administered according to the World Health Organization recommendation [11], and the response was rapid. Diarrhoea progressively reduced, vomiting disappeared, and oral feeding was resumed the day after admission. Blood pressure, heart rate, and respiratory rate returned to normal values within 24 h as it was for diuresis. Pre-renal failure disappeared after 48 h. Weight returned to initial values within one week.

Management of the case was approved by the Ethics Committee of Umbria Region, Perugia, Italy (2018-PED-03), and parents provided informed written consent. The patient’s parents also provided informed consent for the publication of this case report.

### Genetic characterization of the infecting rotavirus (RVA)

Total RNA was extracted from 140 μL of 10% faecal suspensions in distilled water using the Viral RNeasy Mini Kit (Qiagen/Westburg, Segrate, Italy), according to the manufacturer’s instructions. RNA was eluted in 60 μL of RNase-free water and stored at − 80 °C until use. After an initial step of denaturation, the viral RNA was subjected to retro-transcription (RT) using the Invitrogen Superscript III reverse transcriptase kit (Life Technologies, Monza, Italy) with a single cycle at 37 °C for 60 min and 95 °C for 5 min. The obtained DNA was then used as a template for PCR amplification of VP7 (primers Beg9-End9) and VP4 (primers Con3-Con2) segments [12, 13]. The reactions were performed with the Invitrogen Platinum Taq kit (Life Technologies, Monza, Italy), following the manufacturer’s instructions. RVA genotyping was carried out by a multiple semi-nested PCR using a mixture of primers specific for G- and P-types, as previously described for genotype assignment [14]. Retro-transcription and all PCR reactions were performed following slightly modified EuroRota-Net protocols [15].

Since the low amount of DNA obtained after the first round PCR, for nucleotide sequence analysis, a second nested PCR was carried out using VP7-F, VP7-R, VP4-F, and VP4-R primers, resulting in a 881 bp product for VP7 and a 663 bp product for VP4 [16, 17]. Nucleotide sequencing of amplified genes was performed at Eurofins Genomics (Ebersberg, Germany) using primers used for the second PCR. In addition, the amplification and nucleotide sequencing of all the RVA genomic segments was performed. Briefly, the RT-PCR was conducted by using specific primers for each gene segment, and nucleotide sequencing was performed at Eurofins Genomics using the same primers included in the RT-PCR reactions. The nucleotide sequences obtained were analysed and corrected with ChromasPro2.23 software (Technelysium, Queensland, Australia). Nucleotide and amino acid sequence similarity searches were performed using the BLAST (Basic Local Alignment Search Tool) server on the GenBank database of the NCBI (National Center for Biotechnology Information, National Institute of Health, Bethesda, MD).

The assignment of genotypes for all the 11 gene segments was performed with the “RotaC v2.0 - classification tool for rotaviruses group A”. Multiple sequence alignments and phylogenetic tree construction were performed with MEGA6 software [18], applying the maximum-likelihood method and using the Tamura3 + G substitution model. The sequences obtained in this study are available in GenBank (http://www.ncbi.nlm.nih.gov/genbank/) under the accession numbers listed in Table 1.

**Table 1:** Nucleotide sequence identities of rotavirus A strain PG2/2018 compared with the most closely related rotavirus A strains

Gene	GenBank Accession no.	Most closely related genotype (strain)	Nucleotide sequence lenght (position)	Nucleotide sequence identity (%)
VP7	MK158256	G3 (RVA/Human-wt/BRA/IAL-R608/2015/G3P[8])	835 bp (nt 48–882)	99.8
VP4	MK158257	P[8] (RVA/Human-wt/BRA/IAL-R608/2015/G3P[8])	614 bp (nt 186–797)	99.8
VP6	MK780099	I2 (RVA/Human-wt/BRA/IAL-R608/2015/G3P[8])	553 bp (nt 792–1344)	98.5
VP1	MK780096	R2 (RVA/Human-wt/BRA/IAL-R751/2015/G3P[8])	909 bp (nt 28–936)	99.1
VP2	MK780097	C2 (RVA/Human-wt/BRA/IAL-R751/2015/G3P[8])	807 bp (nt 26–832)	99.4
VP3	MK780098	M2 (RVA/Human-wt/BRA/IAL-R751/2015/G3P[8])	877 bp (nt 19–895)	99.3
NSP1	MK780100	A2 (RVA/Human-wt/BRA/IAL-R608/2015/G3P[8])	858 bp (nt 21–878)	99.5
NSP2	MK780101	N2 (RVA/Human-wt/BRA/IAL-R608/2015/G3P[8])	980 bp (nt 35–1014)	99.6
NSP3	MK780102	T2 (RVA/Human-wt/BRA/IAL-R608/2015/G3P[8])	990 bp (nt 40–1029)	99.5
NSP4	MK780103	E2 (RVA/Human-wt/BRA/IAL-R608/2015/G3P[8])	694 bp (nt 49–742)	99.4
NSP5	MK780104	H2 (RVA/Human-wt/BRA/IAL-R608/2015/G3P[8])	764 bp (nt 37–799)	99.6

**Fig. 1:**
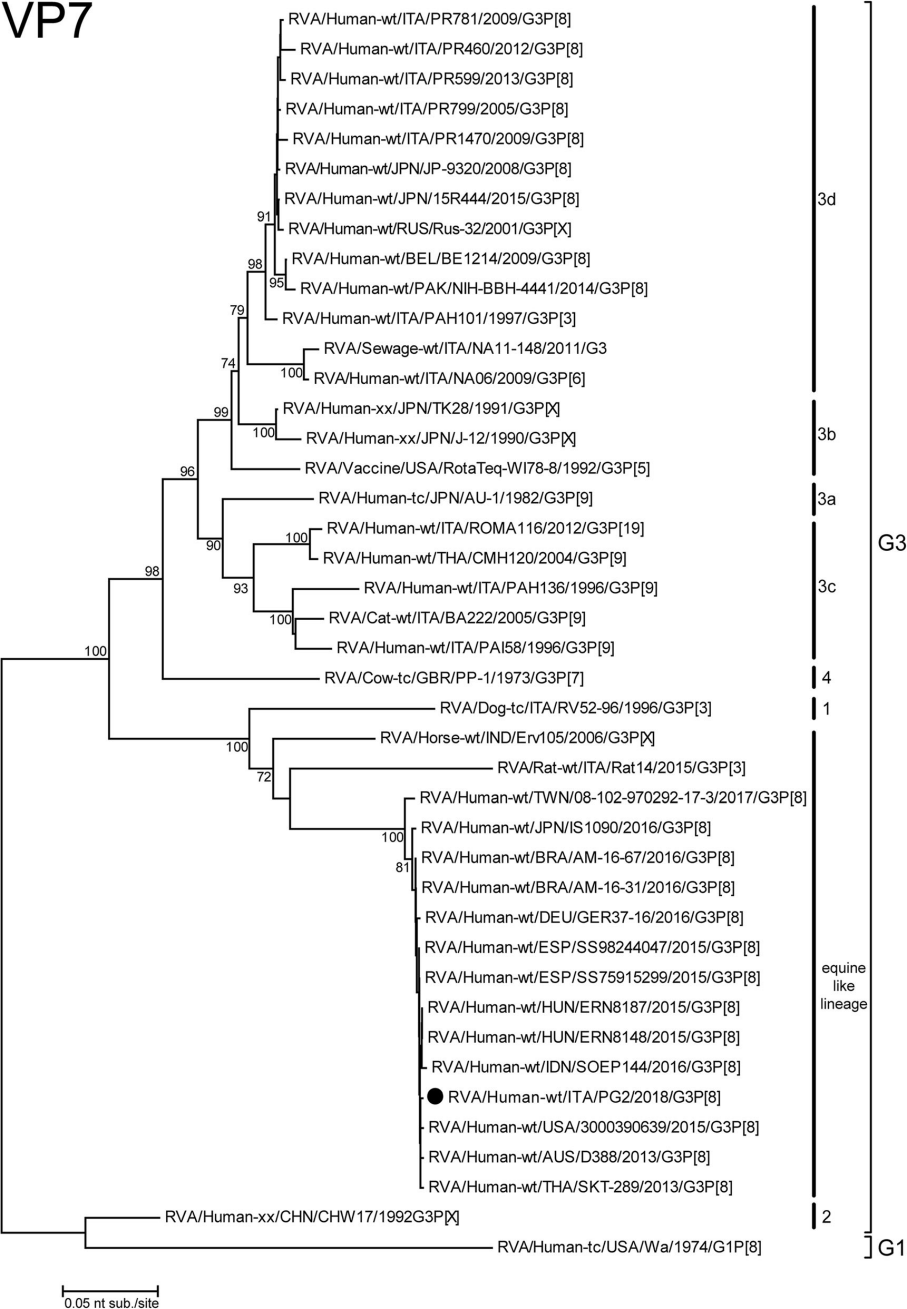
Phylogenetic tree based on the partial ORF of VP7 gene (nt81–910). Italian G3P[8] PG2 strain is highlighted with a filled circle. Trees were built with the maximum likelihood method (ML), and bootstrapped with 1000 repetitions; bootstrap values below 70 are not shown

**Fig. 2:**
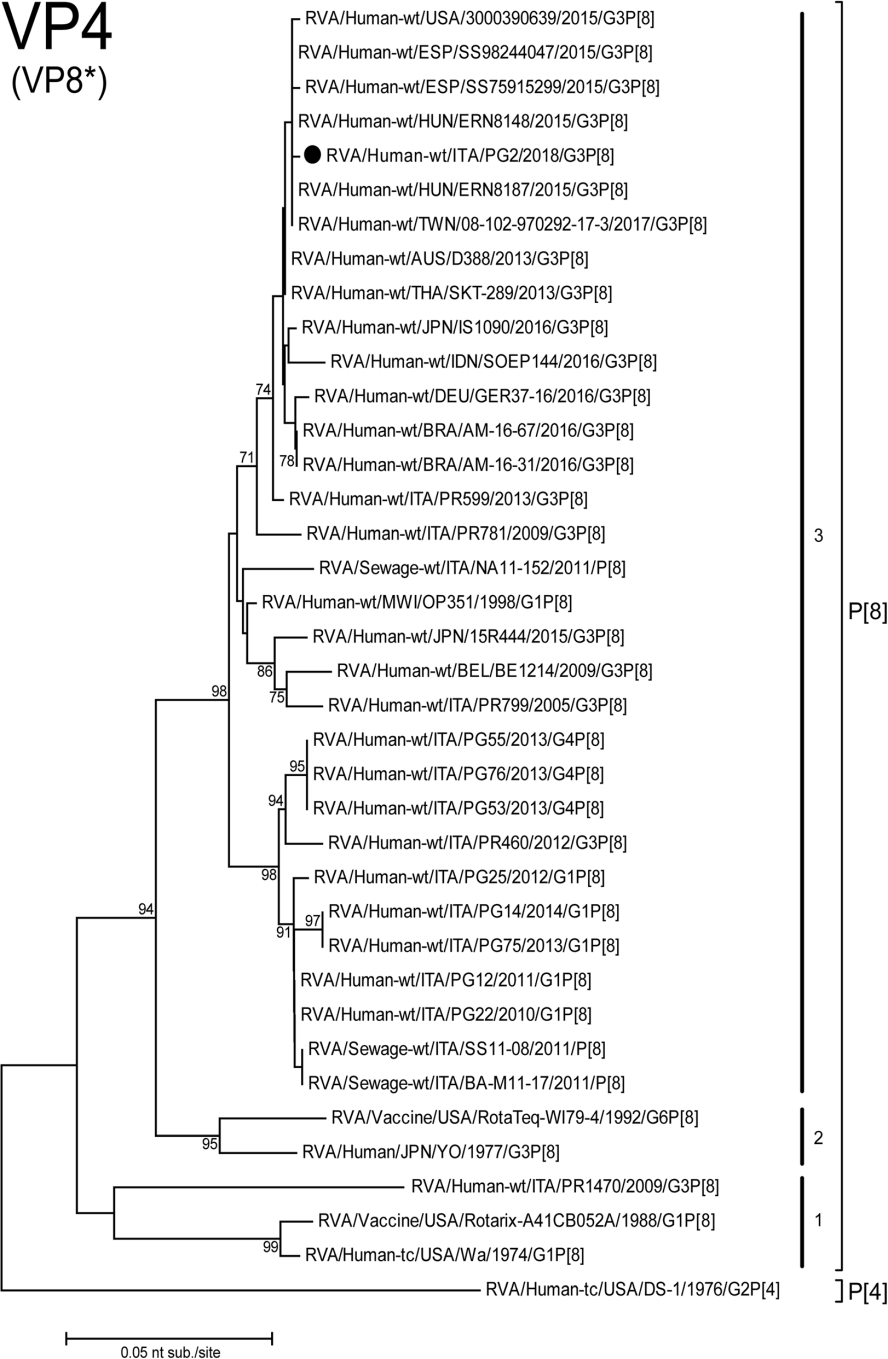
Phylogenetic tree based on the partial ORF of VP4 (VP8*) gene (nt201–789. Italian G3P[8] PG2 strain is highlighted with a filled circle. Trees were built with the maximum likelihood method (ML), and bootstrapped with 1000 repetitions; bootstrap values below 70 are not shown

The phylogenetic trees revealed relatedness between the VP7 and VP4 genes of the PG2 G3P[8] RVA Italian strain and those belonging to recent G3P[8] RVAs detected worldwide. The G3 VP7 belonged to the equine-like G3 lineage and shared the highest nucleotide sequence identity (99.8%) with the previously identified equine-like G3 (Fig. 1). Moreover, it was significantly different from the G3 strains detected previously in Italy and included in the phylogenetic tree within the G3-3c and 3d lineages. The P[8] VP4 revealed a similar clustering pattern as that observed for the VP7. Strain PG2 was closely related to the same strains highlighted by the VP7 analysis, with the P[8] Italian strains previously detected in the same sampling area (in combination with G1 and G4 VP7) grouped separately, despite sharing the same lineage 3 in this case (Fig. 2).

**Fig. 3:**
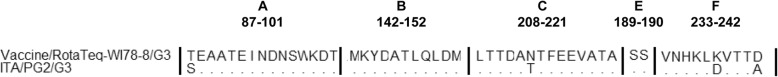
Alignment of the deduced amino acid sequence of the VP7 protein (hypervariable regions **a, b, c, e, f**)**.** The comparison is performed for the G3P[8] PG2 RVA strains included in this study versus the G3 specificity included in the Rotateq anti-RVA vaccine. Dots indicate identities; substitutions are highlighted with the amino acid coding letter

Finally, the PG2 RVA strain clustered in a branch different from that of the VP7 gene of Rotateq® (18.5% nucleotide difference and 7.2% amino acid difference) and in a lineage different from those of the VP4 specificities included in the Rotateq® and Rotarix® vaccines (6.3 and 9.6% nucleotide difference and 5.8 and 10.3% amino acid difference, respectively). The comparison of the deduced amino acid sequences revealed only 4 substitutions (T87A, N213 T, K238D, D242A) across the VP7 epitopes (Fig. 3).

Strain PG2 showed the G3-P[8]-I2-R2-C2-M2-A2-N2-T2-E2-H2 genotype, revealing a complete genotype 2 (DS-1 like) genomic backbone [19]. All the gene segments investigated revealed the highest nucleotide sequence identities (98.5 to 99.8%) with equine-like G3P[8] RVA strains detected in Brazil in 2015 (Table 1).

## Discussion

The case of RVA AGE described here has two peculiarities. It was due to a G3P[8] RVA variant not previously detected in Italy, and it had a very severe course, which is unusual in a school-age child without any known severe underlying problems, such as immunodeficiency. The virus was similar to the human/equine reassortant G3P[8] RVA that has been described in the past as a cause of AGE in several geographic areas, including some European countries [7–9]. However, RVA with these characteristics was never previously detected in Italy. In this country, genetic characterization of G3P[8] RVA isolates identified from 2004 to 2013 did not show any animal/human reassortment, although the emergence of novel lineages through the accumulation of point mutations and reassortments with old human RVA was evidenced [6]. This finding, together with the high identity between PG2 RVA and the already described equine/human reassortant virus, leads to the conclusion that PG2 RVA was imported and is not the consequence of local reassortment. Unfortunately, it is not possible to indicate how the introduction of this virus in Italy could have occurred as the patient did not have unusual travel, dietary, or animal contact.

During 2017, the anti-RVA vaccination was introduced in the list of recommended vaccines to be administered to the paediatric population in Italy. Both Rotarix and Rotateq are available on the market and their administration is not mandatory. The anti-RVA vaccination coverage in Italy is still low (8.41%) and not homogeneous among the different Italian regions (range between 0 and 40%). Data from Umbria region are, to date, not yet available. The emergence of this new G3P[8] raises questions about the effectiveness of current RV vaccines, since the emergence of equine-like G3P[8] in association with a complete DS-1 like genomic constellation represent an epidemiologically important strain that could spread in a population using the monovalent G1P[8] vaccine.As immunity to RVA is believed to be polygenic and include immune responses to RVA antigens other than G and P antigens, substantial modification of RV vaccine efficacy is unlikely to take place, even in cases of high PG2 G3P[8] RVA circulation. On the other hand, both Rotateq® and Rotarix®, have been found able to protect against homotypic and heterotypic rotavirus strains [20–23]. However, the clinical relevance of a new RVA cannot be precisely defined, as mathematical modelling appears to suggest that small reductions in effectiveness of RV vaccines against previously unknown RVAs may significantly alter RVA dynamics over extended periods of time [24–26]. This explains why monitoring of RVA circulation and vaccine effectiveness must be maintained.

Regarding the severity of the case reported here, it cannot be established whether severity was dependent on the virus or host. A potential relationship between RVA strains, mainly G9 strains (P[6] and P[8]), and the development of severe diarrhoea is suggested by some studies but excluded by others [27]. On the other hand, although reassortant equine/human G3P[8] RVA has been associated with severe cases [7], studies evaluating the real impact of this virus in communities where most RVA diarrhoea cases are treated are lacking. However, we cannot exclude the possibility that the host favoured development of severe disease. Host defence against RVA infection is based on several factors, including expression of ABH histo-blood group antigens at the intestinal mucosa where RVA binds and interferon control of RVA replication. Both of these factors can differ between subjects and significantly modulate the severity of RVA [1] infection. Further studies are needed to clarify this problem.

## Conclusion

This case shows the first detection of a reassortant G3P[8] RVA in Italy in an 8-year-old child. It highlights the importance of continuous monitoring of the RVA strains circulating in the Italian paediatric population in order to detect and predict the diffusion of novel viral variants able to spread among the general population.

## Abbreviations

RT: retro-transcription
RV: rotavirus
RVA: group A rotavirus
